# Biotechnological Combination for Co-contaminated Soil Remediation: Focus on Tripartite “Meta-Enzymatic” Activity

**DOI:** 10.3389/fpls.2022.852513

**Published:** 2022-05-06

**Authors:** Maria Tartaglia, Daniela Zuzolo, Alessia Postiglione, Antonello Prigioniero, Pierpaolo Scarano, Rosaria Sciarrillo, Carmine Guarino

**Affiliations:** Department of Science and Technologies, University of Sannio, Benevento, Italy

**Keywords:** rhizosphere, metaorganism, phytoremediation, soil enzymatic activity, soil transcriptomics, *Schedonorus arundinaceus*

## Abstract

Soil pollution is a pressing problem requiring solutions that can be applied without large-scale side effects directly in the field. Phytoremediation is an effective strategy combining plant and root-associated microbiome to immobilize, degrade, and adsorb pollutants from the soil. To improve phytoremediation, it is necessary to think of plants, fungi, and bacteria not as individual entities, but as a meta-organism that reacts organically, synergistically, and cooperatively to environmental stimuli. Analyzing the tripartite enzymatic activity in the rhizosphere is necessary to understand the mechanisms underlying plant–microorganism communication under abiotic stress (such as soil pollution). In this work, the potential of a microbial consortium along with a plant already known for its phytoremediation capabilities, *Schedonorus arundinaceus* (Scheb.) Dumort., was validated in a mesocosm experiment with pluricontaminated soil (heavy metals, PAHs, and PCBs). Chemical analyses of the soil at the beginning and end of the experiment confirmed the reduction of the main pollutants. The microscopic observation and chemical analyses confirmed the greater root colonization and pollutant removal following the microbial treatment. To obtain a taxonomic and functional picture, tripartite (plant, fungi, and bacteria) enzyme activity was assessed using a metatranscriptomic approach. Total RNA was extracted from a sample of rhizosphere sampled considering 2 centimeters of root and soil attached. From the total reads obtained, mRNAs were filtered, and analysis focused on reads identified as proteins with enzymatic activity. The differential analysis of transcripts identified as enzymes showed that a general increase in potential enzyme activity was observed in the rhizosphere after our biotechnological treatment. Also from a taxonomic perspective, an increase in the activity of some Phyla, such as Actinobacteria and Basidiomycota, was found in the treated sample compared to the control. An increased abundance of enzymes involved in rhizospheric activities and pollutant removal (such as dehydrogenase, urease, and laccase) was found in the treated sample compared to the control at the end of the experiment. Several enzymes expressed by the plant confirmed the increase in metabolic activity and architectural rearrangement of the root following the enhancement of the rhizospheric biome. The study provides new outcomes useful in rhizosphere engineering advancement.

## Introduction

The rhizosphere, considered as the soil portion chemically and physically influenced by resident roots activity, varies greatly depending on the characteristics of the plant and microbial community (de Faria et al., 2021). The plant should be understood not as an individual organism, but as a meta-organism in close symbiosis with the soil microbial community with which it has co-evolved. In fact, the substances, produced and secreted by the roots, select and remodel the microbial community (Trivedi et al., 2020). The root exudates contain water, H+ ions, mucilage, and several products, including low molecular weight organic acids, carbohydrates, amino acids, enzymes, and other photosynthates (Vives-Peris et al., 2020). Simultaneously, the selected root-associated microbiome induces feedback in the plant’s performance allowing it to better adapt to adverse abiotic/biotic factors. Plant growth-promoting rhizobacteria (PGPR) increase the availability of nutrients in the soil and promote plant uptake by inducing alterations in the root architecture by promoting the formation of root hairs (Feng et al., 2017; An et al., 2020; Vissenberg et al., 2020). In addition, PGPRs promote plant growth by releasing a complex set of volatile substances, enzymes, and phytohormones and reduce the development of competing pathogens (Vives-Peris et al., 2020).

The total rhizosphere enzyme activity is considered the sum of enzymes present in living root cells, actively released into the soil by the root, released together with cell debris during root cap breakdown, and enzymes of microbial origin, both endophytic and free in the soil (Egamberdieva et al., 2010). Enzymes in the soil catalyze a series of reactions essential for the decomposition of organic matter, soil structure, and fertility and consequently the fitness of resident plant and microbial species. The main enzymes found in the rhizosphere are cellulases, chitinases, amylases, b-glucosidases, dehydrogenases, ureases, phosphatases, arylsulfatases, proteases, lipases, collagenases, pectinases, and others, derived from plants, fungi, or bacteria (Gianfreda, 2015). The soil enzyme activity is closely influenced by the microbial mass present, which in turn is closely dependent on plant biomass and soil characteristics such as organic matter content, management practices, and pollutants. The study of enzymatic activity in the rhizosphere is crucial for assessing the plant–microbiome meta-organism performance, especially under adverse environmental conditions, as the rhizospheric enzymatic setup indicates the soil potential to perform a number of biochemical reactions essential for maintaining good soil structure, fertility, and plant productivity (Liu et al., 2015; Mattarozzi et al., 2020). The methodologies most commonly used today to assess soil enzyme activity do not allow the origin of individual enzymes to be discerned, and thus, the individual contributions of the plant, bacteria, and fungi to be assessed. Considering that most of the bacteria resident in the soil cannot be cultured, it is not possible to isolate them to assess their activity. Furthermore, assessing the enzyme activity directly in the soil matrix gives us a more complete picture of enzyme activity due to the biotic and abiotic interactions occurring.

The accumulation and long persistence of pollutants such as heavy metals (HM), polycyclic aromatic hydrocarbons (PAHs), and polychlorinated biphenyls (PCB) is nowadays a hot topic as the presence of these substances in the soil negatively reflects on the whole trophic chain and can even compromise human health (Zhang et al., 2020). Several strategies are proposed to remove these pollutants from the soil (physical, chemical, biological). One of the most promising of these techniques, both in terms of cost and performance in the field, is phytoremediation, a green, *in situ* strategy that involves exploiting the metabolic capacities of green plants to absorb, remove, and transform pollutants in the soil. Obviously, the contribution of the root-associated microbiome to these complex processes cannot be ignored. The molecular mechanisms underlying phytoremediation are not yet fully understood. However, improving plant conditions by enhancing PGPR communities in the rhizosphere and soil characteristics seems to have a positive impact on phytoremediation performance (Vergani et al., 2017; Simón Solá et al., 2019). The presence of pollutants in the soil alters rhizospheric balances and consequently enzymatic activity. In this work, the performance of a biotechnological approach (R3) in the bio-phytoremediation of a pluricontaminated soil was evaluated, focusing on enzymatic activity in the rhizosphere. The biotechnological approach involved the combined use of bacteria, fungi, and plant (*Schedonorus arundinaceus* (Scheb.) Dumort) in a mesocosms experiment. The choice of the microbial consortium and the plant is supported by previous identification and selection studies. *S. arundinaceus* was selected because of its fast growing feature, phytoremediation performance, its ability to interact with bacteria and fungi at the rhizospheric level, and because it was naturally present on the contaminated site (Li et al., 2013; Steliga and Kluk, 2020; Tartaglia et al., 2022; Zuzolo et al., 2022). Regarding the consortium, it was chosen for its predisposition toward the selected plant, its plant growth-promoting (PGP) activities, and its potential in persistent organic pollutants (POPs) dissipation (Guarino et al., 2019b). The consortium originates from previous studies where microbial taxa were previously isolated from polluted soils, identified by molecular analyses, evaluated for their PGP capabilities, and cultured to produce the inoculum (Guarino et al., 2019a). Given the greater removal of pollutants and the greater root colonization observed in *Schedonorus arundinaceus* (Scheb.) Dumort., following the addition of the biotechnological treatment on pluricontaminated soil, the rhizospheric total potential enzymatic activity was evaluated using the metatranscriptomic approach. By means of a functional metatranscriptomics approach, we were able to have a broad look at the potential total enzyme activity in our samples, both at taxonomic and functional level, framing the main pathways involved in plant–microbiome interaction at root level and in phytoremediation.

## Materials and Methods

### Experimental Workflow

**Figure 1:**
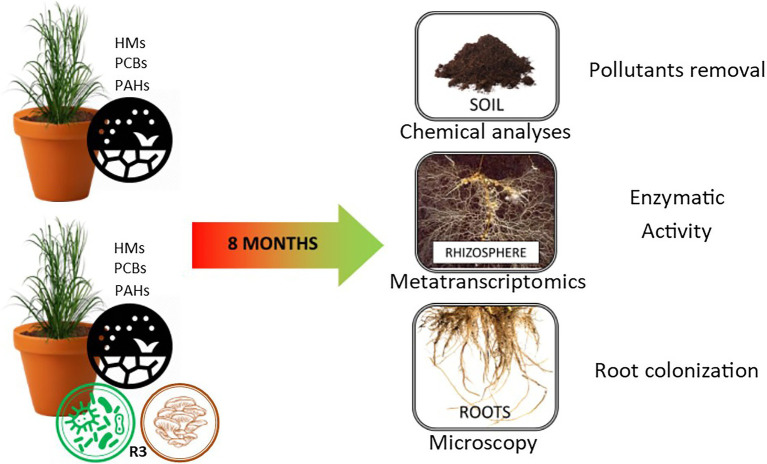
Experimental design.

The mesocosm experiment involved the use of contaminated soil from a polluted site (Bagnoli brownfield site—40°49′30′91 and 40°47′30′North, and 14°9′30′′ and 14°12′0′′ East) characterized by multi-contamination with heavy metals (HMs), polycyclic aromatic hydrocarbons (PAHs), and polychlorinated biphenyls (PCBs) (Table 1). The experiment was conducted in triplicate in 30 kg of soil mesocosms. In the control samples A4R1, a fertilizer mix (150 g of ammonium sulfate NH_4_ 2SO_4_, 150 g of ammonium phosphate NH_4_ 3PO_4_, and 150 g of organic matter with 3 all-organic N P K fertilizing elements per mesocosm) and 20 *S. arundinaceus* seeds (natural *S. arundinaceus* phenotypes grown on site, taken at the end of the reproductive cycle) were added to the pluricontaminated soil; in the treated mesocosms (A2R3), in addition to the fertilizer mix and seeds, twenty grams of microbial consortium *(Rizophagus clarum, Rizophagus intraradicens, Rizophagus irregularis, Rizophagus proliferus, Glomus macrocarpum, Glomus* spp.*, Sordariomycetes*spp., *Claraideoglomus claroideum, Claraideoglomus eutinicatum, Gigaspora marginata, Gigaspora gigantea, Acaulospora* spp., *Burkholderia gladioli, Burkholderia cepacica, Rhodococcus* spp., *Nocardia* spp., *Pseudomonas putida, Pseudomonas fluorescens, Pseudomonas* spp., *Comamonas koreensis, Serratia proteamaculans, Bacillus cereus, Bacillus licheniformis Bacillus megaterium, Bacillus polymyxa, Bacillus subtilis, Bacillus thuringiensis*, and *Paenibacillus polymyxa)* plus 300 g of hay bale inoculated with *Pleurotus ostreatus* (R3 treatment) was used to test the ability of our biotechnological approach to promote the decontamination of soil (Figure 1; Supplementary Table S1). The microorganisms used in our microbial consortium were selected based on previous metagenomic analyses of the pluricontaminated soil under consideration, and based on their PGPR capabilities (Guarino et al., 2019a). Mesocosms were thus prepared and exposed to environmental conditions (the area is characterized by an average annual temperature of 14.4°C and average annual precipitation of about 85.3 mm) for 8 months, after which rhizosphere samples were taken for subsequent analyses.

**Table 1:** Metals and organic pollutants (mg/kg) in control soil (pluricontaminated soil) and in the two treated soils (A4R1, A2R3) at the end of the experiment (8 months).

Pollutant	Contaminated soil	A4R1	A2R3
As	33 ± 2.5^a^	31 ± 1.8^a^	22 ± 1.9^b^
Cd	2.5 ± 0.6^a^	2 ± 0.4^b^	1.3 ± 0.5^c^
Cr	70 ± 2.1^a^	65 ± 1.9^b^	65 ± 1.8^b^
Hg	4.5 ± 0.4^a^	4.3 ± 0.3^a^	1.5 ± 0.3^b^
Ni	22 ± 1.8^a^	21 ± 1.3^ab^	19 ± 1.5^b^
Pb	352 ± 6.7^a^	314 ± 5.5^b^	205 ± 6.2^c^
Cu	82 ± 4.2^a^	80 ± 3.4^a^	72 ± 3.6^b^
Tl	5 ± 1.1^a^	4.6 ± 0.9^a^	3.3 ± 0.8^b^
V	81 ± 3.7^a^	77 ± 4.1^a^	51 ± 3.9^b^
Zn	1,123 ± 12.1^a^	1,093 ± 9.6^b^	652 ± 8.8^c^
Hydrocarbons C < 12	< 5^a^	< 5^a^	< 5^a^
Hydrocarbons C > 12	75 ± 2.3^a^	71 ± 2.1^b^	45 ± 1.8^c^
Benzo[a]anthracene	0.91 ± 0.17^a^	0.77 ± 0.18^ab^	0.58 ± 0.12^b^
Benzo[a]pyrene	1.06 ± 0.11^a^	0.75 ± 0.15^b^	0.71 ± 0.09^b^
Benzo[b]fluoranthene	1.78 ± 0.2^a^	1.52 ± 0.18^b^	1.42 ± 0.13^b^
Benzo[g,h,i]perylene	1.00 ± 0.11	0.81 ± 0.09	0.83 ± 0.10
Benzo[k]fluoranthene	0.70 ± 0.15^a^	0.49 ± 0.17^b^	0.44 ± 0.15^b^
Chrysene	1.54 ± 0.22^a^	1.24 ± 0.19^b^	0.95 ± 0.16^b^
Dibenzo[a,e]pyrene	0.24 ± 0.07	0.21 ± 0.11	0.18 ± 0.09
Dibenzo(A,H)anthracene	0.22 ± 0.09^a^	0.20 ± 0.05^a^	0.15 ± 0.07^b^
Dibenzo[a,h]pyrene	0.54 ± 0.17^a^	0.32 ± 0.11^b^	0.29 ± 0.15^b^
Dibenzo[a,i]pyrene	0.15 ± 0.04^a^	0.10 ± 0.02^b^	0.09 ± 0.03^b^
Dibenzo[a,l]pyrene	0.05 ± 0.02^a^	0.04 ± 0.01^ab^	0.03 ± 0.01^b^
Indeno [1,2,3-c,d] pyrene	1.05 ± 0.15^a^	0.95 ± 0.11^b^	0.83 ± 0.08^b^
Pyrene	1.49 ± 0.21^a^	1.00 ± 0.15^b^	0.93 ± 0.12^b^
∑ PAHs	10.60 ± 2.4^a^	8.20 ± 2.2^b^	7.90 ± 1.9^c^
∑ PCB	7.77 ± 1.7^a^	7.62 ± 1.2^a^	4.62 ± 1.3^b^

Results are presented as mean ± standard error. Different letters indicate significant differences (at *p value* < 0.05, Tukey *post hoc* test) between samples.

### Sampling and Samples Analysis

Soil chemical analyses were performed in triplicate for each mesocosm type to estimate the concentrations of pollutants in the soil at the beginning and at the end of the experiment (8 months). Soil samples were mineralized 24 h at 105°C (CEM, MARSXpress), filtered (0.45 μm polytetrafluoroethylene—PTFE), and the total content of As, Cd, Cu, Pb, Tl, V, and Zn was determined (ICP-OES Varian Inc., VistaMPX), following Zuzolo et al. (2022). Quantification of hydrocarbons in soil (C < 12 and C > 12) was performed according to Guarino et al. (2017). PAHs and PCBs were determined following the US-EPA method 8270D procedure (Guarino et al., 2019b). The USEPA priority PAHs analyzed were: benzo[a]anthracene (BaA), chrysene (Chr), benzo[k]fluoranthene (BkF), pyrene (Pyr), indeno[1,2,3-c,d]pyrene (IP), benzo[a]pyrene (BaP), benzo[b]fluoranthene (BbF), dibenzo[a, h]anthracene (DahA), benzo[g,h,i]perylene (BghiP), dibenzo[a,e]pyrene (DaeP), dibenzo[a,i]pyrene (DaiP), dibenzo[a,h]pyrene (Dahp), dibenzo[a,l]pyrene (DalP), and 31 PCB congeners: dioxin-like PCBs (DL-PCBs: PCB77, PCB81, PCB105, PCB114, PCB118, PCB123, PCB126, PCB156, PCB157, PCB167, PCB169, PCB189, PCB 28, PCB 30, PCB 31, PCB 52, PCB 95, PCB 99, PCB 101, PCB 110, PCB 128, PCB 138, PCB 146, PCB 149, PCB 151, PCB 153, PCB 170, PCB 177, PCB 180, PCB 183, PCB 187).

### Staining and Microscopy of Root Mycorrhizal Fungal Colonization

**Figure 2:**
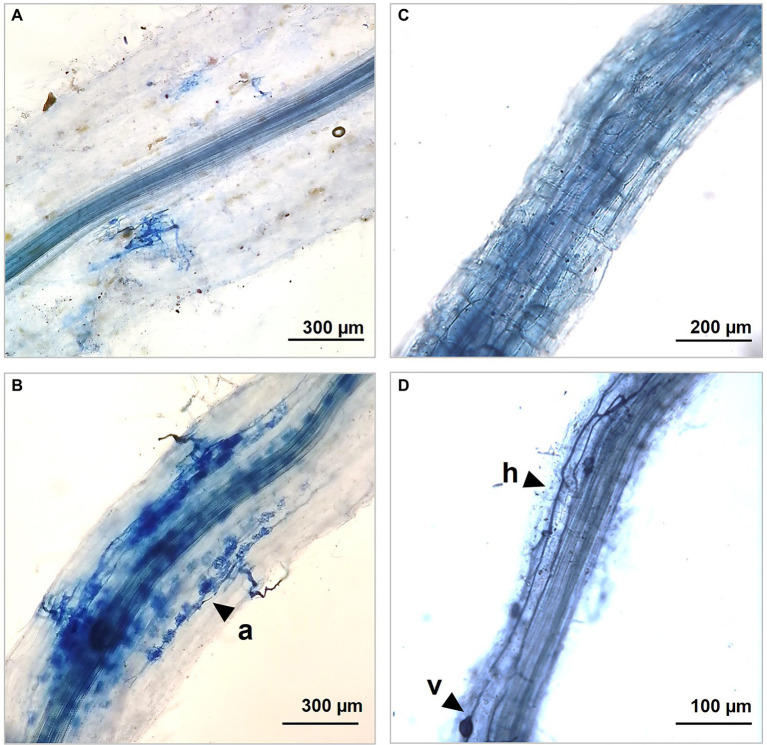
Optic microscopic captions showing different colonization rate in our control A4R1 **(A,C)** and in treated samples A2R3 **(B,D)** at the end of the experiment. R3 treatment induces the development of a wide net of hyphes (h), arbuscules (a), and vescicules (v) that colonize fine *S. arundinaceus* roots developing mutual symbiosis for nutrient exchange between plant and mycorrhizal fungi.

Root portions of *S. arundinaceus* were taken to compare the colonization status. The root sections were fixed in formalin/acetic acid/ethanol (1:1:1) (FAE), gently washed in distilled water, immersed in a 10% KOH solution 15 min at 120°C, rinsed in distilled water and a 3% HCl solution for 20 s, before being stained in a 0.05% trypan blue solution in lactoglycerol (lactic acid/glycerol/water 1:1:3) for 5 min. Excess dye was removed by a step in lactoglycerol at room temperature. To make the measurement scale of mycorrhizal colonization objective, the magnified intersection method (McGonigle et al., 1990) was applied. For each mesocosm (3x A4R1 and 3x A2R3), the roots of 3 plants at the end of the experiment were considered, each of which was divided into 5 subsamples cut into 12 sections of 1 cm and aligned along the long axis of the microscopy slice. The sections, now ready for observation under a light microscope, were placed on slides and the percentage of colonization was assessed using a Nikon Eclipse E600 microscope at 200x magnification. The microscopic observation methodology, already described in Zuzolo et al. (2021) provides that the field of view of the microscope was moved using the stage to make eight constant passes through each fragment to obtain the perpendicular intersection of the vertical crosshair with the root (Zuzolo et al., 2021). Ninety-six intersections were analyzed for each subsample. Whenever the vertical crosshair intersection cut any fungal structure (such as hyphae, vesicles, or arbuscules) annotation increase of one. Each subsample was assessed three times using a new starting point for observation. Hyphal colonization (HC), arbuscules colonization (AC), and vesicular colonization (VC) were calculated and reported as percentages according to McGonigle et al. (1990; Table 2; Figure 2).

**Table 2:** Colonization rate of mycorrhizal structures in *S. arundinaceus* roots at the end of the experiment.

	Sample	
	A4R1	A2R3
Arbuscular colonization (AC)	5.62 ± 1.65%^a^	17.15 ± 2.24%^b^
Vesicular colonization (VC)	4.93 ± 0.52%^a^	9.09 ± 1.86%^b^
Hyphal colonization (HC)	26.31 ± 0.84%^a^	48.88 ± 1.48%^b^

Different letters indicate significant differences (at *p value* < 0.05, ANOVA test) between samples.

### RNA Extraction and Sequencing

To obtain a tripartite metatranscriptomic analysis (plant, fungi, bacteria) with a total enzymatic picture, the apical parts of the roots and 2 cm of adherent soil were collected. The samples were collected and stored at −80°C until further analysis. To extract the total RNA, 2 grams of rhizospheric soil was taken at the end of the experiment, and the Rneasy PowerSoil (Qiagen) extraction kit was used. Extract quantity and quality were verified with the BioAnalyser 2,100. Libraries were prepared using a TruSeq Stranded Total RNA and ribodeplete using the Ribo-Zero Plant Removal Kit. Samples were sequenced using the Novaseq 6,000 Illumina platform with paired-end (PE) readings of 150 bp.

### Metatranscriptomic Profile and Differential Expression Analysis

Raw reads of the Illumina paired-end sequence were filtered for quality by removing low-quality portions while preserving the longest portion of the reads using BBDuck (v. 12_2015). The minimum sequence length was>35 bp and the quality score > 25. Quality control of the filtered sequencing reads was performed using FastQC (http://www.bioinformatics.babraham.ac.uk/projects/fastqc). Residual rRNA sequences were bioinformatically checked and removed by mapping the reads against the SILVA database (SSUParc and LSUParc version 132). The GAIA tool (v2.02) was used to perform the taxonomic profiling of the samples by setting the minimum reading identity>80% and a minimum reading coverage>95%. The taxonomy differential abundance analysis was performed using the DESeq2 R package (version 1.26). A *p* value <0.05, a false discovery rate (FDR) cutoff of 0.05, and a minimum fold change (FC) of 2 were considered thresholds to identify significantly differentially expressed taxa (DE). GAIA has also been used to functionally characterize transcripts and to obtain KEGG (Kanehisa et al., 2012), gene ontology (GO) (Ashburner et al., 2000), and Eggnog (Powell et al., 2012) annotations. The conserved protein domains were identified using Hmmer software (http://hmmer.org/) and Pfam (Finn et al., 2016).

### Data Analyses

The one-way ANOVA was conducted to test group means and assess whether significant difference occurred between the treatments (at a value of *p* < 0.05). For ANOVA significant results, Tukey’s HSD *post hoc* test was run to find out which specific groups’ means differ. ANOVA and *post hoc* analyses were performed using the R *stats* package (R Core Team, 2021). Bubble plot was carried out to depict the identified enzymes profile in the samples, using *stats* (R Core Team, 2021) and *ggplot2* package (Wickham, 2009).

## Results

### Soil Chemical Profile

Chemical analysis of the pluricontaminated soil was performed at the beginning and end of the experiment. The contaminated soil had a high level of inorganic and organic pollutants (Table 1). The levels of As, Cd, Hg, Pb, Sn, Tl, and Zn were above the contamination threshold established by Italian legislation (Legislative Decree 152/2006, 2006). Almost all of the PAH congeners analyzed exceeded the contamination threshold. The concentrations of heavy hydrocarbons (C > 12) and ∑PCB were 75 mg/kg and 7.7 mg/kg, respectively, which denotes very strong pollution considering the contamination thresholds established by Italian legislation (Legislative Decree 152/2006, 2006) limits. Table 1 shows the pollutant concentrations in the initial soil sample used for the experiments, in sample A4R1 taken from the mesocosm treated exclusively with fertilizer mix and *S. arundinaceus* at the end of the experiment, and in sample A2R3 taken from the mesocosm where our microbial background (Supplementary File 1) was added to the fertilizer mix and the plant at the end of the experiment. As can be seen in the table, there is a greater removal of pollutants in sample A2R3, indicating that our microbial treatment had promising potential in phytoremediation of the soil (Table 1). No difference occurred for benzo[g,h,i] perylene and dibenzo[a,e]pyrene.

### Colonization Rate of Mycorrhizal Structures

The colonization rate greatly changes between root without treatment and R3 treatment. Hyphal and arbuscular colonization showed the greatest rate increase after the observation of infected and inoculated roots with R3 as shown in Table 2.

### Filtering and Annotation of Ribosomal RNA

Metatranscriptomic analysis was performed on samples of rhizospheric soil taken at the end of the experiment in the mesocosms with different experimental conditions, in biological triplicate (3xA4R1, 3xA2R3). Sequencing produced an average of 56 million reads per sample, of which 14% were removed after quality control (Supplementary Table S2). Additional filters were applied to remove reads annotated as rRNA.

A large number of reads were classified into proteins, of which an average 250,000 were identified in replicates of A4R1 samples and 400,000 in replicates of A2R3 samples (Supplementary Table S3).

The differential expression analysis of these proteins allowed us to assess which of them were up regulated in the two experimental conditions. For all these proteins, we identified the protein ID from UniRef90, the description of the protein, the gene ontology (GO) IDs associated with the protein, the GO names and, when possible, the enzyme IDs associated with the protein, the enzyme name, and the pathways in which it is involved. Among the differentially expressed proteins, subsequent processing focused only on proteins with identified enzymatic activity.

**Figure 3:**
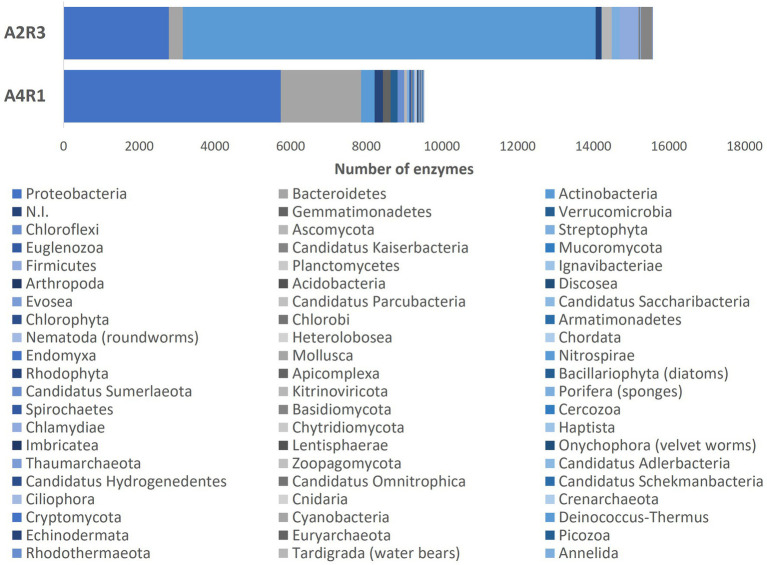
The bars represent the number of differentially expressed transcripts recognized as enzymes identified in the two treatments A4R1 and A2R3, subdivided according to their Phylum.

**Figure 4:**
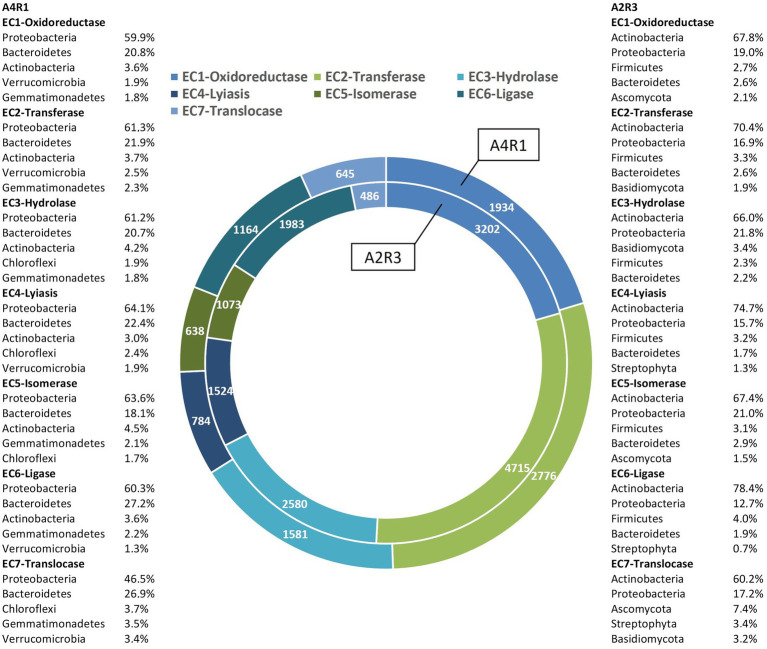
Enzymes up-regulated in A4R1 (tot 9,522) and A2R3 (15,567) subdivided according to their enzyme class (EC1—oxidoreductase, EC2—transferase, EC3—hydrolase, EC4—lyiasis, EC5—isomerase, EC6—ligase, EC7—translocase). For each class, the % of the main Phyla expressing the enzymes are displayed.

As we can see in Figures 3, 4, when comparing the two treatments (A4R1, A2R3), 9,522 up-regulated enzymes were identified in A4R1 and 15,567 up-regulated enzymes in A2R3, considering a significantly increased expression level (over fivefold). Taxonomic analysis showed a substantial difference in terms of Phyla following the addition of the microbial treatment. In our control (A4R1), the highest number of enzymes identified correlated with Proteobacteria, Bacteroidetes, Gemmatimonadetes, Verrucomicrobia, and Chloroflexi. In contrast, in the A2R3 treatment, the majority of enzymes belong to Actinobacteria, followed by Proteobacteria, Firmicutes, Bacteroidetes, Basidiomycota, Ascomycota, and Streptophyta. Figure 4 shows that the differentially expressed enzymes, divided according to the class to which they belong (EC1—oxidoreductase, EC2—transferase, EC3—hydrolase, EC4—lyiasis, EC5—isomerase, EC6—ligase, EC7—translocase), do not vary in relative abundance in the two treatments, but there is a greater total enzyme activity in the A2R3 treatment. The most represented enzyme class is oxidoreductases (1934 enzymes in A4R1 and 3,202 in A2R3), transferases (2,776 enzymes in A4R1 and 4,715 in A2R3), and hydrolases (1,581 enzymes in A4R1 and 2,580 in A2R3). In addition, the 5 most abundant Phyla in percentage are reported for each class. For each class, there was a switch, resulting in enzymes attributable to different Phyla in the two treatments.

**Figure 5:**
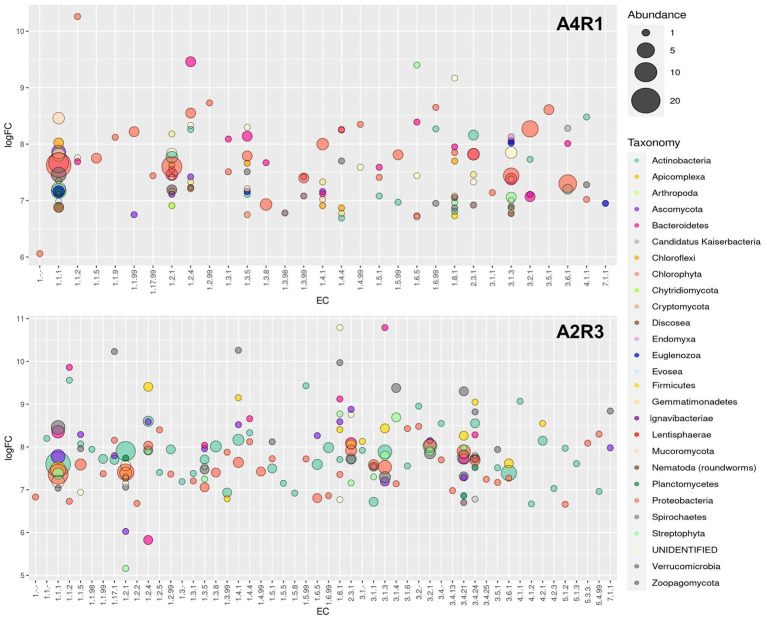
Enzyme overexpression profile. Bubble plot showing the identified enzymes profile in the two sample (A4R1 and A2R3). Data refer to the overexpressed (at least 5 logFC) enzymes of interest. The plot shows the enzyme class (EC) and the average logFC. Enzymes were categorized according to taxonomy (different colors indicate the Taxonomic Phyla) and abundance (different sizes represent the number of identified enzymes belonging to each EC).

From the total number of differentially expressed (DE) enzymes identified, the analysis focused on the main enzymes known in the literature to be involved in polluted rhizosphere activities and plant–microbiome interaction (amylase, arylsulfatase, glucosidase, cellulase, chitinase, chitosanase, dehydrogenase, phosphatase, protease, urease, nitrilase, lipase, pectinase, lecithinase, galactosidase, polyphenol oxidase, laccase). For these enzymes, function, taxonomy, and logFC were considered and show in Figure 5. Figure 5 shows how the abundance of enzymes belonging to the different functional classes varied following the R3 treatment, not only in terms of abundance but also in terms of taxonomic derivation. Actinobacteria appear the main responsible in this switch, shifting the activity of Proteobacteria into the background. The fungal component is also more expressed in A2R3 with a greater presence of enzymes belonging to Ascomycota and Basidiomycota. Enzymes belonging to the Phyla Streptophyta, and to the Poaceae family (Supplementary File S1) are also present to a greater extent in A2R3 than in A1R4, indicating a greater involvement of plants in the enzymatic balance of the rhizosphere following the addition of the R3 treatment. In fact, of the differentially expressed transcripts identified as enzymes and attributable to Streptophyta, 217 are up-regulated in A2R3 and only 63 up-regulated in A4R1. The bulk of the enzymes most highly expressed in both samples belong to EC1—oxidoreductase, and it is evident in Figure 5 that not only are these enzymes generally more abundant in A2R3 but that they belong to a broader taxonomic spectrum than in the control sample. Dehydrogenases are the most represented enzymes in this enzyme class. They are significantly more abundant in A2R3 and are mainly produced by Actinobacteria and Proteobacteria, compared to A4R1 where they are mainly produced by Proteobacteria and Bacteroides (Figure 5). As Figure 5 shows, enzymes belonging to the EC3-hydrolase class are more present in the A2R3 treated sample than in the (A4R1) control. Furthermore, from a taxonomic viewpoint, these enzymes, which in the A4R1 control were mainly derived from Proteobacteria and Bacteroides, are more heterogeneous following our treatment, and in addition to Actinobacteria, the contribution of Basidiomycetes stands out, in particular *P. ostreatus*. A good number of proteases (3.4.21/24), glucosidases (3.2.1.21), laccases (1.10.3.2), and ureases (3.5.1.5) are in fact attributable to the presence of this fungus in the rhizosphere (Figure 5; Supplementary File 1). Also, some enzymes well known in the literature for PAH degradation (path: ec00624) and PCB degradation (path: ec00361), such as Protocatechuate 3,4-dioxygenase (1.13.11.3), Benzene 1,2-dioxygenase (1.14.12.3), Carboxymethylenebutenolidase (3.1.1.45), Alpha/beta hydrolase (3.8.1.5), Haloalkane dehalogenase (3.8.1.5), and Benzaldehyde dehydrogenase (1.2.1.28) are more present in the A2R3-treated rhizosphere sample, highlighting the greater involvement, particularly of Actinobacteria and Proteobacteria, in these pathways (Figure 5; Supplementary File 1).

## Discussion

Soil is a complex ecosystem in which biotic and abiotic components are closely related, and these relationships are amplified at the rhizospheric level where the enzymatic activity is enhanced by the cross talk between plant and associated microbiome (Egamberdieva et al., 2010; Olanrewaju et al., 2019). The plant–microbiome metaorganism responds organically to environmental stimuli, and therefore in phytoremediation strategies, it is necessary to evaluate plants in close symbiosis with the microbiome residing into the rhizosphere (Subramaniam et al., 2016; Olanrewaju et al., 2019; Singh and Prasanna, 2020). To enhance its effects, we selected a pool of microorganisms known to have both a degradation activity and/or act as plant growth-promoting rhizobacteria (PGPR) (Zuzolo et al., 2021). To these in our R3 treatment, a fungus belonging to the Phyla of the Basidiomycota (*P. ostreatus)* was added, known its ability to produce laccases involved in PAHs and PCB degradation (Chun et al., 2019). Chemical analyses, conducted on the pluricontaminated soil at the beginning and end of the experiment, confirmed the increased removal rate promoted by the R3 treatment. Roots microscopic observation confirmed the greater colonization occurred in A2R3. Hyphae, the infecting structures of mycorrhizal fungi, internally explore the root structures and establish symbiotic interactions with the plant through the development of intracellular arbuscules. The exchange of nutrients is the basis of the relationship between plant and mycorrhiza, which develop a network of mycorrhizal structures, capable of extending roots absorbent surface enhancing both nutrient uptake and phytoremediation performances (Walker et al., 2003; Smith et al., 2011). Microbial proliferation and enzymatic activity are the best indicators for assessing the health, stability, and fertility of soil ecosystems (Schloter et al., 2018; Ren et al., 2021). So, established the effect of our biotechnological approach in improving phytoremediation performance through an enhancement of the root-associated microbiome, the focus shifted to the rhizospheric enzymatic activity. The soil enzyme composition is a reflection of resident microbial diversity and abundance, which is closely dependent on the plant species present, influenced by environmental factors (Zuzolo et al., 2022). The soil environment is a source of an immense pool of enzymes. It includes representatives of every enzyme class, i.e., oxidoreductases, hydrolases, isomerases, ligases, liases, and transferases. Soil enzymes perform key functions in the conversion of organic substances and energy and in the interactions between the biotic components of the soil (Burns et al., 2013; Vives-Peris et al., 2020). In our samples, a generally higher enzyme transcript abundance was observed following R3 treatment (microbial consortium + *P. ostreatus*), showing increased tripartite (plant–fungi–bacteria) rhizospheric activity. The oxidoreductases include dehydrogenases (DHO), which are key enzymes in the biological oxidation of soil organic matter through the transfer of hydrogen from the organic substrate to inorganic acceptors. Their enzymatic activity is an essential part of respiratory metabolism, the citrate cycle, N metabolism, and in general in the secondary metabolite biosynthesis. Dehydrogenases are not found free in the soil and directly reflect the oxidative metabolic activity of soil microbes (Butterfield et al., 2016; Deshpande and Nirmalnath, 2016). Consequently, the up-regulation of this pool of enzymes in the sample treated with our biotechnological combination (A2R3), coupled with the microscopic evidence, indicates a general increased rhizosphere microbial activity and suggests a consolidated associations root-microorganism (Gianfreda, 2015; Zuzolo et al., 2021). The increase in enzymes activities at the rhizospheric level is due not only to the stimulation of root-related microbes, but also to the release of enzymes from the root or from lysis of root cells, thus a highly integrated microorganism–plant association (Kantè et al., 2021).

As seen in the literature in PAHs-contaminated soils, there is an increase in DHO activity in the rhizosphere, and so an increase in the organic pollutant degradation, and this effect is strengthened in the presence of resident plants (Alam et al., 2010). The significantly higher abundance of these enzymes in A2R3 indicates improved soil health as a result of enhanced rhizosphere bioactivity and pollutants (both organics and inorganics) decrease, promoted by the metaorganism. The lytic enzymes active in the rhizosphere (mainly proteases, lipases, collagenases, amylases, cellulases, nitrilases, ureases, glucosidases) are essential for hydrolyzing a wide range of polymeric components and have a well-known function in the literature in inhibiting the development of pathogens in the soil, especially fungal pathogens (Yu et al., 2011). Our data show that the presence of lytic enzymes is over-expressed in the R3-treated rhizosphere, indicating better beneficial outcomes both on plant protection and plant growth promotion (Yu et al., 2011). Most of the enzyme activity found in the plant is involved with the metabolism of amino acids, carbohydrates, and lipids, and is up-regulated in the A2R3-treated sample. In the literature, it is well known that this increased metabolic activity of the plant positively reflects, through the root exudates, on the soil biome (Walker et al., 2003). The role of ethylene, and its precursor ACC, in bacterial colonization of the root is also recognized. This phytohormone modulates the plant immune response and alters the architecture of the root. This gaseous hormone in fact inhibits the elongation of the root and instead stimulates the production of root hairs. Root hairs are essential for the acquisition of nutrients and for the plant–soil–resident microbiome interaction. Ethylene promotes their development through still little-known molecular mechanisms; however, ROOT HAIR DEFECTIVE proteins are well-documented positive regulators of root hair development and are under the control of phytohormones, especially ethylene (Feng et al., 2017; Vissenberg et al., 2020). In the A2R3 treated sample, both the key enzyme in ethylene production from its ACC precursor (ACC oxidase) and the ROOT HAIR DEFECTIVE 3 enzyme are strongly up regulated, suggesting a positive stimulation of root hair production in the *S. arundinaceus* roots as a result of the R3 treatment. The altered root architecture of *S. arundinaceus*, under the same experimental conditions, was already observed in a previous study by scanning electron microscopy (SEM) (Zuzolo et al., 2022). The metabolic activity of *P. ostreatus* in pluricontaminated soils is well documented and is essential for the oxidation of the most recalcitrant PAHs. Ureases can be free and enzymatically active in the soil after cell lysis. Free ureases in the soil have a direct activity on roots, promoting the release of root exudates, influencing pH, improving mineral availability for the plant. Such a stimulated increase in plant biomass has positive feedback on the soil microbial mass proliferation (Qu et al., 2019). Following our R3 treatment, we observed an increased number of ureases produced by both Actinobacteria, Proteobacteria, and Basidiomycetes. Although the pluricontaminated soil undoubtedly represents a hostile environment for the plant, the close and mutually beneficial communication at the rhizospheric level may allow the resulting metaorganism to better cope with stress and increase phytoremediation performance, in agreement with data from our previous studies (Zuzolo et al., 2022). The presence, predominantly and sometimes even exclusively (as in the case of *P. ostreatus*-related laccases), of many enzymes involved in organic pollutant degradation pathways demonstrates that the observed removal of organic pollutants is an effect, both direct and indirect, of our biotechnological treatment.

## Conclusion

Understanding the metabolic processes performed by microbes and plants in polluted niches is necessary to manipulate the meta-organism underlying the phytoremediation. Knowledge about the taxonomic composition and functions of the rhizospheric biome is still incomplete due to the soil matrix heterogeneity and the impossibility of culturing most of the microorganisms present. Investigating how selective pressure from pollutant compounds has resulted in the evolution of microbial communities allows us to exploit new enzymatic processes capable of modifying or degrading the recurrent compounds. The use of a metatranscriptomic approach allowed us to have a potential picture of the tripartite enzymatic activity among plants, fungi, and bacteria. This could explain the higher pollutant removal rate we observed in our samples following the R3 biotechnology application. In fact, if the chemical analysis of the soil and microscopic analysis of the root have confirmed the colonization of the root and phytoremediation; the analysis of the differential expression of enzymatic proteins in rhizospheric soil has allowed us to identify which are the main actors, and what are their contributions, on the stage of phytoremediation. Associating taxonomy with function could be the key to understanding and engineering the rhizosphere. As a future perspective, it would be desirable to validate the potential enzymatic activity found, going down to a deeper level of analysis, and therefore through a rhizospheric metaproteomic analysis, made more complex by the difficult extraction of proteins from the soil necessary for the close interaction between proteins and humic substance.

## Data Availability Statement

The datasets presented in this study can be found in online repositories. The names of the repository/repositories and accession number(s) can be found at: National Center for Biotechnology Information (NCBI) BioProject database under accession number PRJNA799745.

## Author Contributions

CG and RS: conceptualization and methodology. MT and DZ: formal analysis. MT: writing-original draft and validation. All authors contributed to the article and approved the submitted version.

## Funding

This research was funded by the Italian MIUR PRIN2017BHH84R.

## Conflict of Interest

The authors declare that the research was conducted in the absence of any commercial or financial relationships that could be construed as a potential conflict of interest.

## Publisher’s Note

All claims expressed in this article are solely those of the authors and do not necessarily represent those of their affiliated organizations, or those of the publisher, the editors and the reviewers. Any product that may be evaluated in this article, or claim that may be made by its manufacturer, is not guaranteed or endorsed by the publisher.

## Supplementary Material

The Supplementary Material for this article can be found online at: https://www.frontiersin.org/articles/10.3389/fpls.2022.852513/full#supplementary-material
