# Supplementary material for: Biotechnological Combination for Co-contaminated Soil Remediation: Focus on Tripartite “Meta-Enzymatic” Activity
Source: Front Plant Sci. 2022 May 6;13:852513. doi: 10.3389/fpls.2022.852513 (PMC9121008; doi:10.3389/fpls.2022.852513)
Supplement: Supplementary file 1 [file Table_1.DOCX]

| **Supplementary Table 1.** Treatment of mesocosms produced in triplicate from 30 kg of contaminated soil   \| **A4R1** \| **A2R3** \| \| --- \| --- \| \| 30 kg Contaminated Soil \| 30 kg Contaminated Soil \| \| 20 *S. arundinaceus* seeds \| 20 *S. arundinaceus* seeds \| \| 150 g NH4 2SO4, 150 g NH4 3PO4, 150 g of organic matter with 3 all-organic N P K \| 150 g NH4 2SO4, 150 g NH4 3PO4, 150 g of organic matter with 3 all-organic N P K \| \|  \| 20g of microbial consortium (*Rizophagus clarum, Rizophagus intraradicens, Rizophagus irregularis, Rizophagus proliferus, Glomus macrocarpum, Glomus spp, Sordariomycetes spp, Claraideoglomus claroideum, Claraideoglomus eutinicatum, Gigaspora marginata, Gigaspora gigantea, Acaulospora spp, Burkholderia gladioli, Burkholderia cepacica, Rhodococcus spp, Nocardia spp, Pseudomonas putida, Pseudomonas fluorescens, Pseudomonas spp. Comamonas koreensis, Serratia proteamaculans, Bacillus cereus, Bacillus licheniformis Bacillus megaterium, Bacillus polymyxa, Bacillus subtilis, Bacillus thuringiensis and Paenibacillus polymyxa*) \| \|  \| 300 g of hay bale inoculated with *Pleurotus ostreatus* \|   **Supplementary Table 2.** The number of reads before and after quality check was reported as well as the number of reads mapping to the SILVA database (No rRNA reads) and the number and percentage of cleaned reads (whitout rRNA). | | | | | |
| --- | --- | --- | --- | --- | --- | --- | --- | --- | --- | --- | --- | --- | --- | --- | --- | --- | --- |
|  |  |  |  |  |  |
| **Sample_ID** | **No of reads before QC** | **No of reads after QC** | **No of reads rRNA *** | **No of cleaned reads** | **% of cleaned reads*** |
| A4R1_rep1 | 60.746.691 | 51.441.276 | 5.087.784 | 46.353.492 | 90,11% |
| A4R1_rep2 | 58.927.289 | 50.072.272 | 7.118.820 | 42.953.452 | 85,78% |
| A4R1_rep3 | 61.454.549 | 52.027.840 | 7.091.510 | 44.936.330 | 86,37% |
| A2R3_rep1 | 49.135.434 | 41.422.794 | 20.672.098 | 20.750.696 | 50,09% |
| A2R3_rep2 | 58.706.541 | 50.170.294 | 18.834.352 | 31.335.942 | 62,46% |
| A2R3_rep3 | 50.581.459 | 43.461.132 | 21.060.791 | 22.400.341 | 51,54% |
| * (relative to trimmed reads) | |  |  |  |  |

**Supplementary Table 3**. Number of reads classified by Gaia Function module. This table is reporting the number of reads instead of the number of read pairs.

| **Sample** | **Number of reads classified to proteins** | **Number of proteins identified** |
| --- | --- | --- |
| A4R1_rep1 | 7,586,399 | 238,049 |
| A4R1_rep2 | 6,511,376 | 266,524 |
| A4R1_rep3 | 7,043,100 | 272,213 |
| A2R3_rep1 | 8,220,346 | 466,434 |
| A2R3_rep2 | 11,551,638 | 427,296 |
| A2R3_rep3 | 7,062,621 | 323,191 |
