# Supplementary figures and images for: Biotechnological Combination for Co-contaminated Soil Remediation: Focus on Tripartite “Meta-Enzymatic” Activity
Source: Front Plant Sci. 2022 May 6;13:852513. doi: 10.3389/fpls.2022.852513 (PMC9121008; doi:10.3389/fpls.2022.852513)

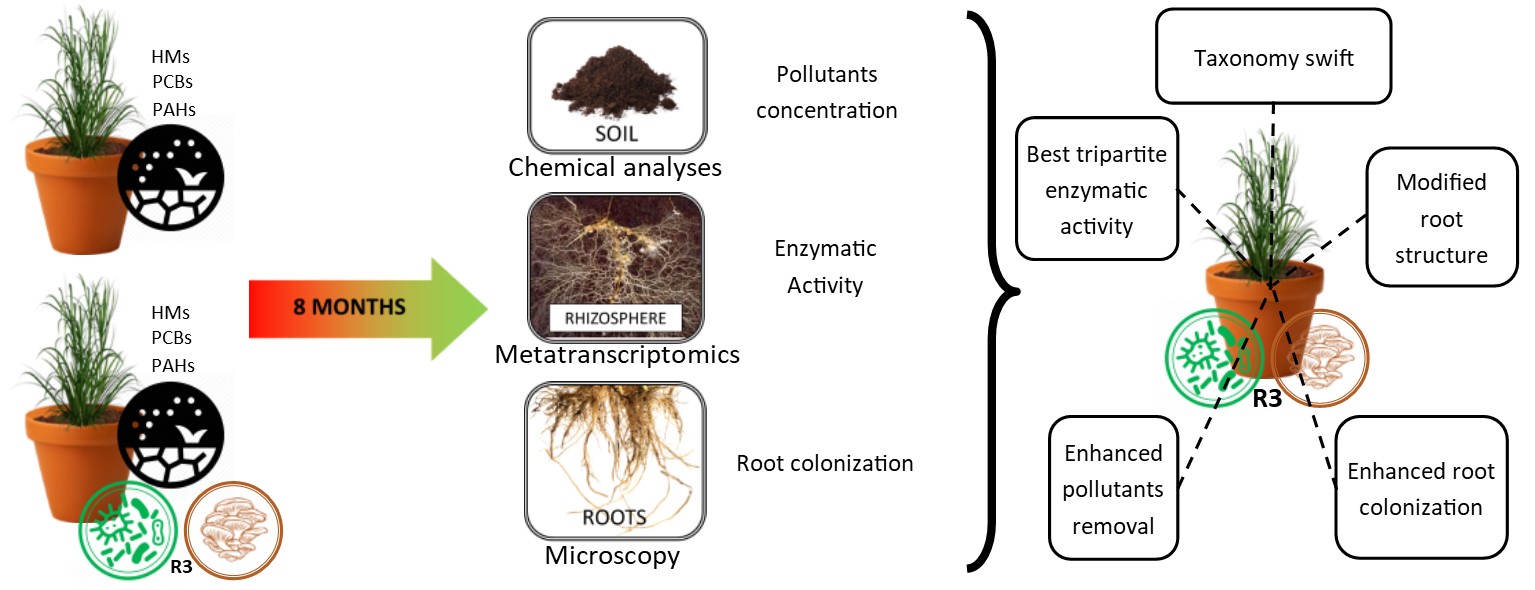

Supplement: Supplementary file 3 [file Image_1.JPEG]
